# Differential Neuroprotective Activity of Two Different Grape Seed Extracts

**DOI:** 10.1371/journal.pone.0014575

**Published:** 2011-01-24

**Authors:** Keishi Narita, Masashi Hisamoto, Tohru Okuda, Sen Takeda

**Affiliations:** 1 Department of Anatomy and Cell Biology, Interdisciplinary Graduate School of Medicine & Engineering, University of Yamanashi, Yamanashi, Japan; 2 The Institute of Enology and Viticulture, University of Yamanashi, Yamanashi, Japan; Tokyo Institute of Psychiatry, Japan

## Abstract

Glutamate excitotoxicity is one of the major events that takes place during various neurotoxic injuries such as brain ischemia. We prepared grape seed extracts, from two different varieties, containing high amounts of polyphenols but little resveratrol. Their neuroprotective effects were investigated using primary culture of neonatal mouse hippocampal neurons treated with an excitotoxic concentration of glutamate. Koshu, a white, local variety of *V. vinifera*, alleviated the acute inactivation of Erk1/2 and dendrite retraction in cultured hippocampal neurons exposed to a toxic concentration of glutamate (1.0 ng/ml). By contrast, Muscat Bailey A, a red, hybrid variety (Muscat Humburg × Bailey), failed to show any neuroprotective effect. Unlike brain-derived neurotrophic factor and other neuroprotective cytokines, Koshu extract did not induce Akt phosphorylation. Koshu extract also augmented neuron survival rate 24 hours after glutamate toxicity. The comparison of polyphenols between the two samples by liquid chromatography/time-of-flight mass spectrometry demonstrated that Koshu had higher amounts of low molecular weight polyphenols along with several Koshu-specific procyanidin oligomers. These data suggest the presence of high affinity molecular targets for polyphenols in hippocampal neurons, which induce neuroprotective effects in a manner different from BDNF, and the importance of low molecular weight polyphenols and/or procyanidin oligomers for neuroprotection.

## Introduction

Polyphenols are a large group of chemical compounds possessing phenolic groups, which exhibit antioxidant properties. Thousands of naturally occurring polyphenolic compounds are believed to exist in plants, including vegetables and fruits. Epidemiological studies suggest that increased consumption of vegetables and fruits has beneficial effects on human health, for which the natural polyphenols have been considered as the major responsible elements [1]. For example, the relatively low incidence of heart attacks in the French population is attributable partly to red wine consumption, which is rich in polyphenols [2]. So far, the best-characterized polyphenols in this context are the grape stilbene resveratrol and the tea flavonoid epigallocatechin gallate (EGCG). It has been shown that polyphenols not only act as simple antioxidants, but also interact with specific molecular targets directly and indirectly within the cell, modulating distinct cell signaling pathways to each other [3], [4], [5]. For example, Balasubramanian and Eckert demonstrated that EGCG stimulated keratinocyte differentiation, whereas apigenin and curcumin exhibited the opposite effect [6]. Similarly, Yazawa *et al*. reported that neuroprotective mechanisms of curcumin, tannic acid and (+)-catechin are different from each other [7]. Resveratrol has been shown to exhibit various beneficial biological effects, such as protective effects against cardiovascular diseases, anti-inflammatory or anticarcinogenic activities. Resveratrol has also been shown to pass the blood brain barrier (BBB) and exert neuroprotective effect, and therefore its use for the prevention and treatment of both acute and chronic neurodegenerative diseases, such as cerebral ischemia and Alzheimer's disease, has been investigated [8], [9], [10]. Considering the wide structural diversity of polyphenolic compounds, one could expect that there would be many other polyphenols and/or their various combinations that would induce unique biological effects beneficial to our health.

Grape seeds, which are removed as pomace during the winemaking process, are also known to be a rich source of polyphenols. Unlike the skin, grape seeds are known to contain little resveratrol [11]. In the present study, in order to investigate the potential neuroprotective effects of polyphenols other than resveratrol, we chose grape seed extracts (GSEs) from two different varieties. Koshu (KOS) is a white, Yamanashi-native variety of *V. vinifera*, which is said to have been imported from China to Japan ∼800 to 1,200 years ago [12]. Muscat Bailey A (MBA) is a red hybrid. By analyzing early changes of cultured hippocampal neurons in response to an excitotoxic concentration of glutamate, we demonstrate that the KOS but not MBA possess neuroprotective activities. Furthermore, by comparison of the polyphenol contents between the two GSEs, we also demonstrate differences in the polyphenol composition between KOS and MBA.

## Results

### Koshu grape seed extract protects Erk1/2 phosphorylation in cultured hippocampal neurons during glutamate excitotoxicity

**Figure 1:**
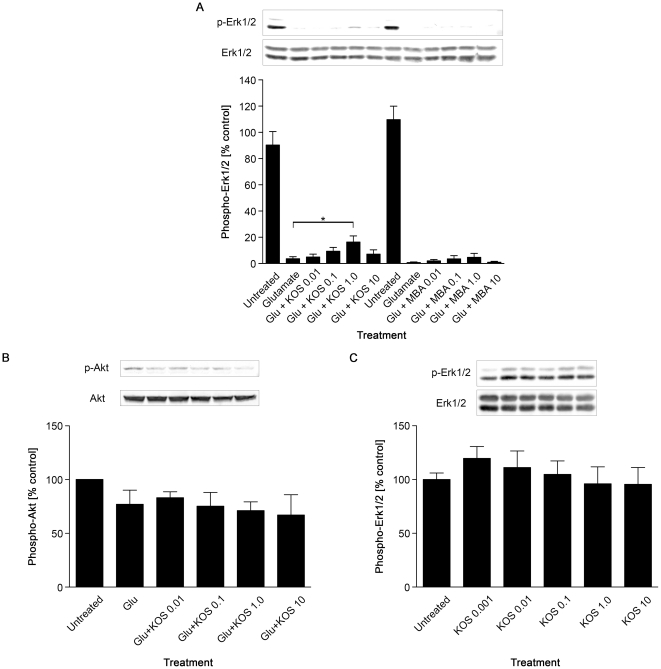
Koshu GSE alleviates glutamate-induced inactivation of Erk1/2 in cultured hippocampal neurons. (A) *Top*, detection of phosphorylated Erk1/2 in cultured hippocampal neurons by western blot. Neurons were treated with 50 µM glutamate alone or in the presence of indicated concentrations (in ng/ml) of KOS or MBA for 30 min. The immunoblot for total Erk1/2 shows equal loading. *Bottom*, quantification of phospho-Erk1/2 levels by image analysis (*n* = 5). The relative values to the untreated controls (set to 100%) are shown. *, *P*<0.05 versus untreated controls. (B) Hippocampal neurons were treated with 50 µM glutamate with or without indicated concentrations (in ng/ml) of KOS GSE for 30 min, and 200 µg of total protein were loaded in each lane for western blot analyses for phospho- and total Akt. The basal Akt phosphorylation appeared slightly diminished upon glutamate insult. KOS GSE did not show any protective effect on basal Akt phosphorylation levels. (C) The effect of KOS GSE alone on Erk1/2 phosphorylation in cultured hippocampal neurons. Cells were treated with indicated concentrations (in ng/ml) of KOS GSE for 30 min. *Top*, representative western blot images of phospho- and total-Erk1/2. *Bottom*, a quantification of the phospho-Erk1/2 levels by image analysis (*n* = 3).

To evaluate neuroprotective effects of KOS and MBA GSEs, cultured mouse hippocampal neurons were treated with an excitotoxic concentration of glutamate in the presence of various concentrations of GSEs, and the levels of Erk1/2 phosphorylation were quantified by western blotting. Treatment of cultured hippocampal neurons with 50 µM glutamate alone for 30 min caused a severe reduction of Erk1/2 phosphorylation to almost undetected levels (Figure 1A). However, when cells were treated with 50 µM glutamate in the presence of different concentrations of KOS GSE, a modest, but significant, protection of Erk1/2 phosphorylation was observed at 1.0 ng/ml (Figure 1A). Higher concentrations of KOS GSE did not alleviate Erk1/2 dephosphorylation. Indeed, an overnight treatment with >100 ng/ml KOS GSE alone appeared to be cytotoxic, as it altered the morphology of cultured hippocampal neurons and reduced cell number (data not shown). By contrast, MBA GSE failed to protect Erk1/2 phosphorylation during glutamate excitotoxicity with all the concentrations tested (Figure 1A). KOS GSEs had no effect on Akt phosphorylation in cultured neurons (Figure 1B). We also performed another control experiment to see the effect of KOS GSE *per se* on ERK phosphorylation, in the absence of glutamate injury. Treatment with different concentrations of KOS GSE alone for 30 min did not show any significant effect on Erk1/2 phosphorylation in cultured hippocampal neurons (Figure 1C). These data collectively demonstrate that KOS GSE specifically protects Erk1/2 activity in cultured hippocampal neurons during excitotoxic stress, at a narrow concentration range (1.0 ng/ml).

### Koshu GSE also protects dendrite processing of hippocampal neurons during glutamate excitotoxicity

**Figure 2:**
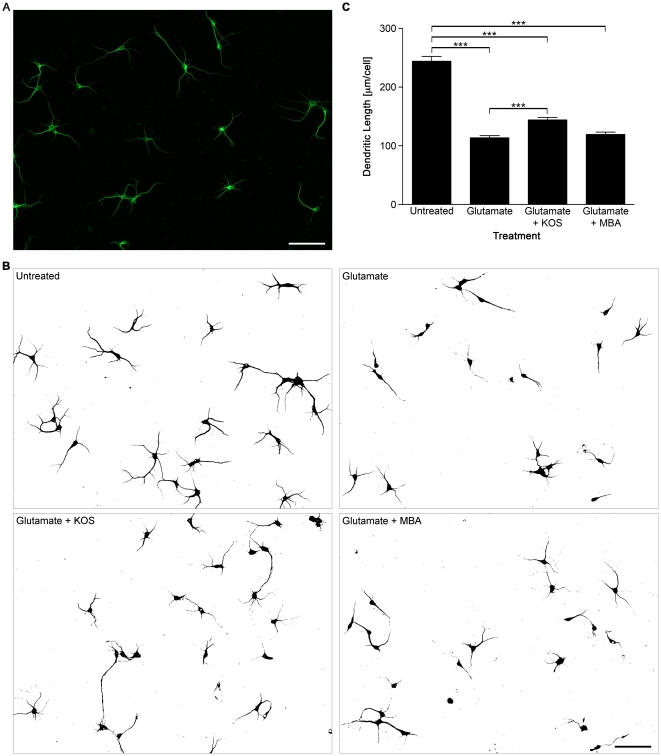
Koshu GSE protects dendrite processing of cultured hippocampal neurons exposed to a toxic concentration of glutamate. (A) Representative image of untreated hippocampal neurons at 8 DIV immunostained for MAP2. Bar, 100 µm. (B) Representative binary images of cultured hippocampal neurons immunostained for MAP2 with no treatment or after a 30 min treatment with 50 µM glutamate alone, 50 µM glutamate plus 1.0 ng/ml KOS, or 50 µM glutamate plus 1.0 ng/ml MBA. Bar, 100 µm. (C) Quantification of the mean dendrite length by morphometric analysis (*n* = 10). ***, *P*<0.001 versus untreated controls.

Next, we validated the protective effect of GSEs based on neuronal morphology. Under normal conditions, cultured hippocampal neurons at 8 days *in vitro* (DIV) extend long, highly branched dendrites and are heavily connected to each other, as demonstrated by immunostaining for microtubule-associated protein 2 (MAP2) (Figure 2A and B). However, when neurons were treated with glutamate for 30 min, stressed neurons exhibited morphological changes. The deleterious effects of glutamate on neuronal morphology were recognized as low as 10–20 µM, in which some MAP2-positive dendrites showed an irregular punctate staining, indicative of neuronal damage (data not shown). When neurons were exposed to 50 µM glutamate, the length and branching of dendrites in neural networks were severely reduced (Figure 2B). A morphometric analysis indicated that the mean dendrite length of stressed neurons was reduced by 50% as compared to the untreated control (Figure 2C). When cells were treated with 50 µM glutamate in the presence of 1.0 ng/ml KOS GSE, the dendritic network connecting distant neurons, as well as the number of branches appeared to be better preserved compared to cells treated with glutamate alone (Figure 2B). Treatment with KOS GSE also alleviated the reduction in mean dendrite length significantly (Figure 2C). On the other hand, 1.0 ng/ml MBA GSE did not show any protective effect on dendrite arborization during glutamate excitotoxicity, which was consistent with the Erk1/2 phosphorylation data. These data indicated that KOS, but not MBA GSE, protected dendritic arborization of hippocampal neurons exposed to excitotoxic concentrations of glutamate.

### Neuroprotective effects of Koshu GSE after glutamate treatment

**Figure 3:**
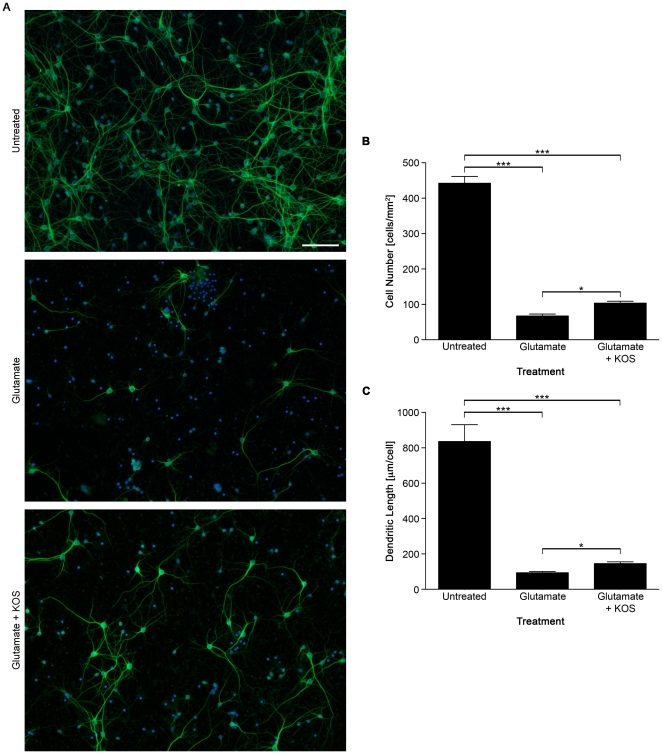
Koshu GSE augments cell survival of cultured hippocampal neurons after excitotoxic treatment. Hippocampal neurons plated at 2.0×10^5^ cells/cm^2^ were treated with 50 µM glutamate in the presence or absence of 1.0 ng/ml KOS GSE for 10 min and allowed to recover in conditioned medium for 24 hr. (A) Representative images of hippocampal neurons after treatment. Bar, 100 µm. (B) The mean density of MAP2-positive neurons surviving 24 hr after glutamate treatment (*n* = 8). ***, *P*<0.001; *, *P*<0.05. (C) Quantification of the mean dendrite length by morphometric analysis (*n* = 8). ***, *P*<0.001; *, *P*<0.05.

To determine the neuroprotective effect of KOS GSE on neurons exposed to glutamate, we performed a cell survival experiment. Hippocampal neurons were treated with 50 µM glutamate in the presence or absence of 1.0 ng/ml KOS GSE for 10 min, and allowed to recover in conditioned medium for 24 hr. Cells were fixed and immunostained for MAP2 (Figure 3A) to identify surviving neurons and analyze dendrite length. In samples treated with glutamate alone, approximately 15% of neurons survived (Figure 3B). The survival rate increased to 23% following addition of KOS GSE during the glutamate insult (Figure 3B). Morphometric analysis also demonstrated that the mean dendrite length in neurons treated with KOS GSE was longer than those treated with glutamate alone (Figure 3B). These data indicated that KOS GSE not only protected dendritic arborization, but also augmented cell survival of cultured hippocampal neurons after the glutamate insult.

**Figure 4:**
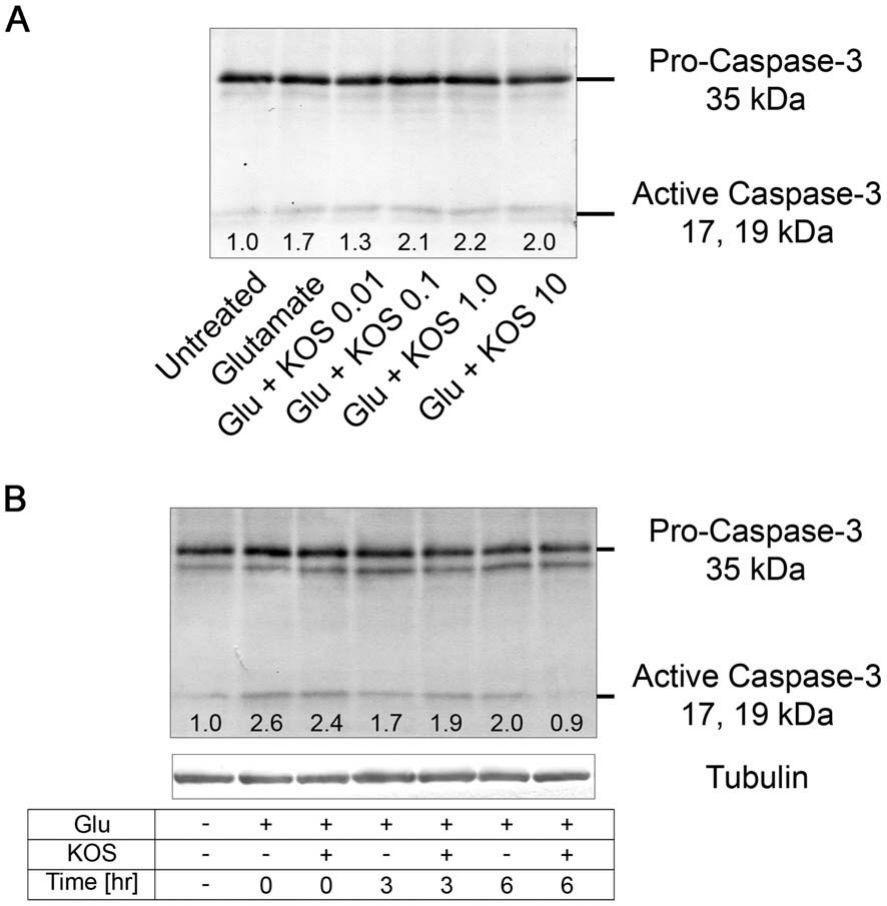
Effect of Koshu GSE on active caspase-3. (A) Hippocampal neurons treated with 50 µM glutamate alone or in the presence of the indicated concentrations (in ng/ml) of KOS GSE for 30 min were analyzed for active-caspase-3 by Western blot. Numbers below the active caspase-3 bands indicate their relative intensities quantified by densitometric analysis. (B) Hippocampal neurons treated with 50 µM glutamate in the presence or absence of 1.0 ng/ml KOS GSE for 30 min and incubated in normal culture medium for 0, 3, or 6 hr were analyzed for active-caspase-3 by Western blot. Numbers below the active caspase-3 bands indicate their relative intensities quantified by densitometric analysis.

To gain insight into the molecular mechanisms of neuroprotection by KOS GSE, we investigated the levels of active caspase-3 by western blot. When levels of active caspase-3 were examined immediately after a 30 min treatment with 50 µM glutamate in the presence of varying concentrations of KOS GSE, we observed an increase in the levels of active caspase-3 in all samples treated with glutamate, with no correlation with KOS GSE concentrations (Figure 4A). However, 6 hr after the insult, the levels of active caspase-3 reduced back to normal levels in neurons treated with 1.0 ng/ml KOS GSE, whereas neurons treated without KOS GSE showed a sustained activation of caspase-3 (Figure 4B). These data suggest that the apoptosis pathway may be a target of KOS GSE.

### Comparison of polyphenolic contents between two grape seed extracts

**Figure 5:**
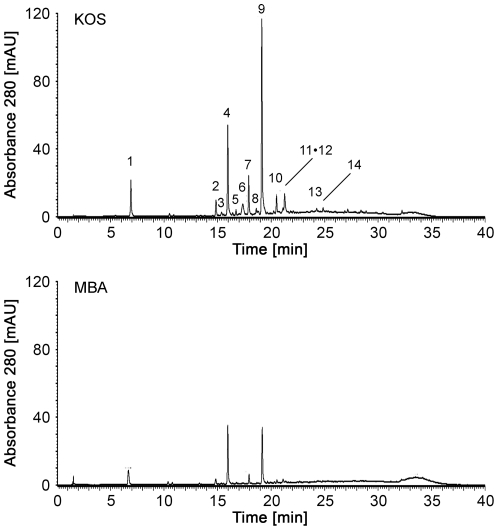
Typical liquid chromatography profile of Koshu and MBA grape seed extracts. Polyphenols in the eluate were detected by absorbance at 280 nm. The peaks were assigned by TOF-MS as shown in Table 1.

Quantification of total phenolics in both GSEs by the free radical scavenging assay demonstrated that the amounts were 85.5% and 73.7% for KOS and MBA, respectively. To investigate the difference in composition of polyphenolic contents between KOS and MBA GSEs, samples were subjected to liquid chromatography/time-of-flight mass spectrometry (LC/TOF-MS) analysis as described in the Materials and Methods. Four relatively small molecular weight polyphenols, catechin, epicatechin, and procyanidins B_1_ and B_2_, were detected in the GSE of both KOS and MBA (Figure 5 and Table 1). In addition to these four major polyphenols, gallic acid was also detected as a common phenolic compound. Furthermore, several extra peaks were observed only in the KOS GSE eluent from the liquid chromatography column (Figure 5). Most of these compounds unique to KOS were identified as procyanidin oligomers by the following time-of-flight mass spectrometry (Table 1, asterisks). Resveratrol was not detected in either GSE.

**Table 1:** LC/TOF-MS data of phenolic compounds.

No.	*t*R [min]	Compound	*m*/*z* [M-H]^-^	*m*/*z* of in-source fragment ions
**1**	6.91	Gallic acid	169.0141	125.0232
**2**	14.86	Procyanidin B_1_	577.1332	407.0693, 289.0701, 245.0778
3*	15.37	Procyanidin dimer	577.1282	407.0728, 289.0678, 245.0655
**4**	15.95	(+)-Catechin	289.0716	245.0789, 203.0715
5*	16.72	Procyanidin trimer	865.1968	713.1505, 695.1157, 577.1189, 407.0724, 289.0743, 245.0564
6*	17.35	Procyanidin dimer	577.1344	407.0749, 289.0713, 245.0683
**7**	17.91	Procyanidin B_2_	577.1341	407.0733, 289.0683, 245.0772
8*	18.63	Procyanidin dimer	577.1326	407.0753, 289.0712, 245.0707
**9**	19.13	(−)-Epicatechin	289.0706	245.0792, 203.0666
10*	20.51	Procyanidin trimer	865.1982	713.1497, 695.1391, 577.1267, 407.0772, 289.0677, 245.0544
11*	21.26	Procyanidin dimer gallate	729.1464	577.1216, 407.0760, 289.0700, 245.0597
12*	21.31	Procyanidin tetramer	1153.2642	1001.2333, 983.2046, 865.1931, 577.1223, 407.0764, 289.0703, 245.0601
13*	24.26	Epicatechin-3-*O*-gallate	441.0824	289.0706, 245.0644, 169.0143
14*	24.85	Procyanidin dimer	577.1276	407.0717, 289.0692, 245.0713

Asterisk indicates those only detected in KOS GSE.

In KOS GSE, the most abundant molecule was epicatechin, followed by catechin and procyanidins B_1_ and B_2_. In MBA GSE, the contents of catechin and epicatechin were similar to each other. Total amounts of these compounds were 154 and 60 mg/g GSE for KOS and MBA, respectively (Table 2). The remainder of the total weight was most likely made up of high molecular weight polyphenols such as proanthocyanidins. These data indicated that KOS and MBA GSEs contain different composition in polyphenolic compounds, and that KOS GSE showed a higher content of small molecular weight polyphenols and procyanidin oligomers than MBA.

**Table 2:** Polyphenol contents of grape seed extracts (mg/g of dry weight) from KOS and MBA.

	Catechin	Epicatechin	Procyanidin B_1_	Procyanidin B_2_	Total
**KOS**	41.00±1.78	85.01±0.38	9.29±0.09	18.82±0.09	154
**MBA**	26.57±0.29	24.83±0.24	3.57±0.04	4.70±0.04	60

## Discussion

### Neuroprotective effect of Koshu grape seed extract against glutamate excitotoxicity

Excitotoxicity and oxidative stress are major events induced by brain ischemia and following reperfusion, leading to neuronal damage [13]. Namura *et al*. reported a severe reduction in Erk1/2 phosphorylation in the mouse hippocampus during experimental forebrain ischemia [14]. Our present study demonstrated that KOS GSE alleviated the glutamate-induced decline of phosphorylated Erk1/2 levels in cultured hippocampal neurons (Figure 1) and protected the dendrites from retraction during a 30-min glutamate insult (Figure 2). This observation is consistent with previous observations by Almeida *et al*., reporting that brain-derived neurotrophic factor (BDNF) increased Erk1/2 phosphorylation in cultured hippocampal neurons and protected cells from glutamate-induced apoptosis [15]. Limatola *et al*. also demonstrated that fractalkine CX3CL1 protected hippocampal neurons from glutamate excitotoxicity through the activation of the Erk1/2 [16]. Interestingly, these molecules are reported to induce the phosphorylation of Akt. However, unlike these cytokines, Koshu GSE did not activate the Akt signaling pathway in hippocampal neurons (Figure 1). These data suggest that the molecular targets of Koshu GSE in hippocampal neurons are somewhat different from those of BDNF or CX3CL1, implying the possibility of developing a new target of ischemic treatment. We also examined the cell survival rate and dendrite length of cultured neurons 24 hr after glutamate treatment to confirm the neuroprotective effect of KOS GSE (Figure 3).

The molecular mechanisms of neuroprotection by KOS GSE remain to be clarified. Since our present study demonstrated that KOS GSE alone had no significant effect on ERK1/2 phosphorylation (Figure 1C), we speculate that it might inhibit the activity of glutamate receptors or their direct modulators to alleviate the neuronal condition during glutamate excitotoxicity. Interestingly, Gao *et al*. demonstrated that *trans*-resveratrol at concentrations of 10–100 µM inhibits postsynaptic glutamate receptors in hippocampal neurons, with NMDA receptors being more sensitive than AMPA receptors [17]. Inhibition of ERK phosphatases [18], [19] may also account for the observed increase in ERK phosphorylation in cells stimulated with glutamate in the presence of the KOS GSE. From another standpoint, our western blot analysis for active caspase-3 demonstrated that KOS GSE did not inhibit the early onset of glutamate-induced caspase-3 activation, but reduced levels 6 hr after the insult (Figure 4). Because caspase-3 is an effector caspase, whose activation occurs in the execution phase to kill cells indiscriminately, our data may not suggest that KOS GSE protects neurons by reversing apoptosis. Our current speculation to explain this observation is that the cultured hippocampal neurons were heterogenous in terms of their sensitivity or response kinetics to glutamate, and KOS GSE protected the relatively resistant or slowly responding population from apoptosis.

### Effective concentration of Koshu grape seed extract in hippocampal neuron

We demonstrated that KOS GSE exhibited its neuroprotective effect at 1.0 ng/ml (Figures 1 and 2). The effective concentration appeared to be extremely low compared to numerous previous reports showing various biological effects of polyphenols within µg/ml ranges. For example, Suenaga *et al*. demonstrated that 3–10 µg/ml resveratrol upregulated TGF-β2 expression in A549 human lung epithelial cells [20]. Fujishita *et al*. also reported that the effective concentration of KOS GSE to induce IL-6 expression in astrocytes was 30–100 µg/ml [11]. Considering the high concentration of polyphenols reported previously, these effects were not mediated by the specific receptors for polyphenols. However, Han *et al*. demonstrated that radiolabeled resveratrol bound to the rat brain plasma membrane fraction with an apparent affinity of 220±45 nM (∼50 ng/ml) and various polyphenols competed against specific resveratrol binding, suggesting that a common receptor binding site was enriched in the brain plasma membrane [21]. Similarly, Campos-Esparza *et al*. also reported that neuroprotective concentrations of mangiferin and morin were 100 nM (∼42 ng/ml for mangiferin and ∼32 ng/ml for morin) [22]. There might be neuron-specific plasma membrane proteins that would show high affinity to polyphenols, including the molecules present in KOS GSE. In this respect, the present study proposes a new paradigm in molecular mechanisms of polyphenol to neurons.

### Difference between Koshu and MBA grape seed extracts

The KOS GSE has been shown to contain catechin, epicatechin, procyanidin B_2_ and proanthocyanidins [11]. In the present study, we identified the same molecular components (Figure 5, Tables 1 and 2), demonstrating the reliability of our data. The comparison of KOS and MBA demonstrated a marked difference in the amount of small molecular weight polyphenols (Table 2). This appeared to be different from the reports by Kammerer *et al*. and Guendez *et al*., demonstrating no significant differences in polyphenol contents between red and white grape seeds [23], [24]. Whether the difference between KOS and MBA were constant regardless of vintages or vineyards will be addressed in the future. Nevertheless, our titration data to protect Erk1/2 activity during glutamate excitotoxicity indicated that MBA failed to show any positive effect in all concentrations tested (Figure 1). Therefore, the difference between KOS and MBA in the content of total phenolics or amount of major polyphenols would be insufficient to explain their difference in neuroprotective activities.

Interestingly, our LC/TOF-MS data demonstrated the presence of KOS-specific peaks, most of which were procyanidin oligomers (Figure 5 and Table 1). Procyanidin oligomers, including procyanidins B_1_ and B_2_, are formed mostly by condensation of catechins, epicatechins and other flavonoids. The structures of procyanidin oligomers are diverse, since the combinations of monomer units, the positions and types of linkage, as well as the degree of condensation reaction have many possibilities [25]. Several reports exist demonstrating that the degree of grape seed polyphenol oligomerization affects its biological activity to scavenge the reactive oxygen species [26], [27]. Among the various KOS-specific procyanidin oligomers, some might have favorable structures to interact with putative neuron-specific receptors to exhibit neuroprotective properties. Similar to our notion, Takahashi *et al*. demonstrated that grape seed procyanidin oligomers exhibit higher growth-promoting activity than the monomer or other flavonoids toward murine hair epithelial cells *in vitro* and *in vivo*, suggesting that the specific effect might be associated with structure [28].

The composition of polyphenols and relative ratios in the presence of minor polyphenols may also induced synergistic effects in KOS GSE. Taking into account that various polyphenols compete for the specific resveratrol binding site [21], this idea may explain the current results. This may also explain why KOS GSE as low as 1.0 ng/ml could provide a neuroprotective effect.

### Therapeutic perspectives

Our present study demonstrated that KOS GSE protects cultured hippocampal neurons against glutamate-induced insults of acute Erk1/2 inactivation and dendrite retraction. It will be of great interest to test whether the active components of KOS GSE directly influence the cellular activity of neurons or improve the cellular conditions via glia-mediated mechanisms [11]. Nevertheless, as brain ischemia induces damage to the BBB, an effective concentration could be easily achieved by intravenous administration. For example, Hwang *et al*. reported that oral administration of South Korean GSE 30 min before or after experimental forebrain ischemia protected hippocampal neurons from oxidative DNA damage [10], suggesting the possibility of GSE for therapeutics. GSE may also be beneficial for other excitotoxic brain insults, such as epilepsy [29].

## Materials and Methods

### Materials

KOS grape seed was obtained from local winemakers in Yamanashi in 2005. MBA grape seed was obtained from the experimental vineyard of the University of Yamanashi in 2005. The freeze-dried GSE powder was prepared as described previously [11]. In both preparations, total polyphenol accounted for >90% of net dry weight (data not shown). The resulting powder was dissolved in dimethyl sulfoxide(DMSO)/ethanol/H_2_O (50∶25∶25) and used for cell culture experiments. Total phenolics in both GSEs were quantified as described previously [30].

Poly-L-lysine, papain, deoxyrobonuclease (DNase), cytosine arabinofuranoside (Ara-C), L-glutamate and mouse anti-MAP2 antibody (M9942) were from Sigma-Aldrich. Leibovitz L-15 medium, Neurobasal-A medium, B27 supplement, as well as alkaline phosphatase- or Alexa Fluor 488-conjugated secondary antibodies were obtained from Invitrogen. The cell strainer with 100 µm nylon mesh was from BD. Trichloroacetic acid (TCA) and the Protein Assay Bicinchoninate Kit were from Nakalai Tesque. Immobilon-P membrane was from Millipore. Rabbit anti-Erk 1/2 (#9102), anti-phospho-Erk1/2 (#9101), anti-Akt (#9272), anti-phospho-Akt (Ser473; #4051) and anti-Caspase-3 (#9662) antibodies were from Cell Signaling Technology. Nitro blue tetrazolium chloride (NBT) and 5-Bromo-4-chloro-3-indolyl phosphate, toluidine salt (BCIP) were from Roche Diagnostics.

### Cell culture

Full details of the mouse study were approved by the Institutional Animal Care and Use Committee at the University of Yamanashi (Approval number: 19–92). All mice were handled according to the Guide for the Care and Use of Laboratory Animals. Primary culture of hippocampal neurons were prepared from neonatal murine pups (C57Bl/6J mice, Charles River Laboratories) as described by Berbari *et al* [31] with modifications. Briefly, hippocampal tissue isolated from newborn mice (P1) was treated with 0.375 mg/ml papain and 50 µg/ml DNase in Leibovitz L-15 medium containing 0.2 mg/ml bovine serum albumin for 15 min at 37°C. The tissue was then washed three times with Neurobasal-A medium containing B-27 and gently triturated by 10 strokes with a 1000-µL micropipette followed by another 20 strokes with a 200-µL micropipette. After centrifugation at 200×*g* for 5 min at 4°C, hippocampal neurons were resuspended in Neurobasal-A/B-27 containing 5 µg/ml DNase, passed through a cell strainer with 100 µm mesh, and plated at 1.0×10^5^ cells/cm^2^ on culture dishes or glass coverslips precoated with poly-D-lysine. At 3 DIV, Ara-C was added to a final concentration of 5 µM to prevent glial proliferation.

To assess neuroprotective effects of GSEs, cultured hippocampal neurons at 8 DIV were treated with 50 µM glutamate for 30 min, in the presence or absence of various concentrations of GSEs. The volume of DMSO/ethanol/H_2_O solvent was set to 0.5% (v/v) of medium. Following the treatment, cells were quickly fixed with ice-cold 10% (v/v) TCA and used for western blot or immunostaining.

### Western blot

Equal amounts of protein (20 µg/lane, or 200 µg/lane to detect the basal Akt phosphorylation) were separated by electrophoresis on 10% (v/v) or 5–20% (v/v) SDS poly-acrylamide gels and transferred to Immobilon-P membranes. To inhibit phosphatase activities, 1 mM sodium fluoride was added to the sample lysis buffer. After blocking with Tris buffered saline with Tween 20 (TBST) containing 10% (w/v) non-fat dry milk for 1 h at room temperature (RT), the blots were incubated with the primary antibodies diluted 1∶500 in the blocking buffer and incubated overnight at 4°C. The blots were then washed with TBST and incubated with alkaline phosphatase-conjugated secondary antibody diluted 1∶500 in the blocking buffer at RT for 1 h. After washing with TBST, the immuno-reactive bands were visualized using NBT/BCIP substrates.

### Immunocytochemistry and morphometric analysis

Neurons fixed on glass coverslips were permeablized with −20°C methanol, blocked with 10% (w/v) skim milk in phosphate buffered saline (PBS) for 30 min, and incubated with anti-MAP2 antibody (dilution: 1∶200) overnight at 4°C. After washing with PBS, the samples were incubated with the Alexa Fluor 488-conjugated secondary antibody (1∶200) for 30 min at RT, washed again with PBS and mounted on glass slides with 4′,6-diamidino-2-phenylindole (DAPI). The stained cells were investigated under an Olympus IX71 microscope equipped with UPlanSApo 10× objective and DP72 cooled CCD color camera, and the images were taken with DP2-BSW software and stored as TIFF files. Fields were chosen randomly to ensure objectivity of sampling. However, as the cell density affects the dendrite length significantly, and the high cell density images tend to underestimate the mean dendrite length due to overlap, images with a cell density of ∼50±15 cells/mm^2^ were used for the following morphometric analysis.

The mean dendrite length was quantified using ImageJ software as follows, in which an image analysis method for tumor vessel architecture [32] was applied with modifications. First, the acquired 24-bit color images were split into red, green, and blue channels, and the green channel containing the information of the Alexa Fluor 488 chromophore was binarized to black and white using a common threshold level that would best separate the dendrite structure and background. The resulting binary images were then processed further with the skeletonize command to reduce all the binalized dendrites to black lines with a single pixel width. The total area of skeletonized dendrites in each image, corresponding to the total dendrite length, was counted by the particle analysis command, and the total number of neuron in each image was counted based on nuclear staining with DAPI. Finally, the mean dendrite length was calculated by dividing the total dendrite length by the total cell number.

### Liquid chromatography/time-of-flight mass spectrometry analysis

For LC/TOF-MS analysis, freeze-dried GSEs (10 mg) were dissolved in 10 ml 50% (v/v) aqueous DMSO. The solution was centrifuged at 12,000×*g* for 10 min, and filtered through 0.2 µm PTFE filter.

LC/TOF-MS analyses were carried out using an Acquity Ultra Performance liquid chromatography (UPLC) system (Waters) linked simultaneously to both a PDA 2996 photo diode array detector (Waters) and a Waters LCT premier XE mass spectrometry (Waters), equipped with a electrospray ionization (ESI) source. Two µl of sample solution was injected into an Acquity UPLC column (HSS T3, 1.8 µm, 2.0 mm×100 mm, Waters), which was maintained at 40°C. Linear gradient analysis was used with mobile phase A, 0.1% (v/v) formic acid, and mobile-phase B, acetonitrile with a flow rate of 0.2 ml/min. Elution condition was performed as follows: 0.0 min, 1% (Mobile-phase B, v/v); 3.0 min, 1%; 40.0 min, 60%; 42.0 min, 100%; 44.0 min, 100%; 44.1 min, 1%; 50.0 min, 1%. All of the above experiments were replicated three times each. The eluate was introduced into a PDA detector (scanning range 200-600 nm, resolution 1.2 nm) and subsequently into ESI source in negative mode. The acquired *m*/*z* was from 100 to 2,000. The ionization parameters were capillary voltage, 2.8 kV; cone voltage, 60 V; the desolvation gas flow, 500 l/h; the desolvation temperature, 350°C; and source temperature, 120°C. For the dynamic range enhancement (DRE) lockmass, a solution of leucine-enkephalin (Sigma-Aldrich) of 20 ng/ml was infused through the Lock Spray probe at a flow rate of 5 µL/min. Peak assignments were carried out based on the retention times, their Abs. 280 nm and MS spectrum.

### Statistical analysis

All values were expressed as the mean ± SEM. Differences between two groups were compared using two-tailed *t* test. Differences between multiple groups were analyzed with one-way ANOVA followed by Bonferroni post hoc test to compare selected pairs. Results were considered significant at *P*<0.05.
